# Spatial ligand–receptor circuits in osteosarcoma: immune resistance in bone and lung niches

**DOI:** 10.3389/fimmu.2026.1932933

**Published:** 2026-10-20

**Authors:** Yijian Qiu, Shuai Deng, Junfeng Zhou, QiLin Tan, Yiyi Pan, Enchen Li, Ke Ren, Qingsong Wu

**Affiliations:** 1 School of Sports Medicine and Health, Chengdu Sport University, Chengdu, China; 2 Department of Orthopedics, the Affiliated Sports Hospital of Chengdu Sport University, Chengdu, China

**Keywords:** cancer-associated fibroblasts, ligand–receptor signaling, macrophages, osteosarcoma, pulmonary metastasis, spatial immunology

## Abstract

Osteosarcoma (OS) has shown limited sensitivity to single-agent immune checkpoint blockade. The distribution of immune cells within bone tumors and pulmonary metastases suggests that defective tumor-cell recognition, restricted tissue access and local suppression may contribute to this resistance. This mini-review examines ligand–receptor communication between malignant cells, myeloid cells, fibroblasts and vascular or bone-associated compartments, with attention to the anatomical and experimental settings in which each interaction was studied. Human tissue profiling identifies distinct tumor interiors, myeloid-rich interfaces and fibrocollagenous boundaries, whereas perturbation studies test selected mechanisms within these compartments. GD2-dependent suppression of macrophage phagocytosis involves human SIGLEC7 or murine Siglec-E and downstream SHP2 signaling. CXCL14–integrin α11β1 signaling links OS cells to fibroblast activation and pulmonary niche formation. A separate metabolic circuit couples oxidized-lipoprotein uptake by CD36-positive fibroblasts to ANGPTL4 secretion and integrin α5β1-dependent CD8 T-cell dysfunction. CD24 and macrophage migration inhibitory factor also have OS-specific functional support, although the receptor dependencies inferred from transcriptomic data require more precise attribution. Bone-remodeling and vascular pathways are considered microenvironmental barriers rather than interchangeable immune checkpoints. We distinguish human spatial observations from cell-culture experiments, engineered bone models and animal studies, and separate experimental immune effects from clinical antitumor activity. We also consider how cell-to-tumor distances, compartment-specific receptor expression and stromal architecture could inform biomarker development. These measurements remain candidates for prospective testing rather than established criteria for treatment selection. Linking tissue localization to receptor engagement and immune function offers a route to testing which circuits sustain disease in bone, which support pulmonary dissemination and which persist across both settings.

## 1 Introduction

Osteosarcoma (OS) is the most common primary malignant bone tumor in children and adolescents, with incidence varying by age and population (1, 2). Surgery and multi-agent chemotherapy remain central to treatment of localized high-grade disease (3, 4). Patients presenting with metastases have substantially poorer outcomes, particularly when complete surgical clearance is not possible (5). The lung is therefore both a major site of treatment failure and an important setting in which to examine immune resistance.

Clinical studies of pembrolizumab-based treatment have shown limited activity in advanced OS (6, 7). Immunogenomic profiling identified low immune infiltration and restricted T-cell receptor clonality in many tumors, alongside adaptive resistance programs in more inflamed lesions (8). Together, these findings point to heterogeneous constraints on immune recognition and tumor-cell killing that extend beyond PD-1 signaling.

Single-cell and spatial studies offer a way to resolve this heterogeneity. Human OS atlases describe malignant differentiation states and regional stromal or immune programs (9, 10). In pulmonary metastases, fibrocollagenous encapsulation and compartmentalized immune populations make total immune-cell abundance an incomplete description of tumor access (11, 12). Ligand–receptor (LR) analysis can connect these observations to candidate signals between malignant and nonmalignant cells. This review examines the resulting mechanisms across bone and lung niches, concentrating on interactions that alter phagocytosis, immune positioning or fibroblast-mediated suppression. **Figure 1** summarizes their anatomical context; **Table 1** separates the evidence for each interaction from its proposed immune consequence.

**Figure 1:**
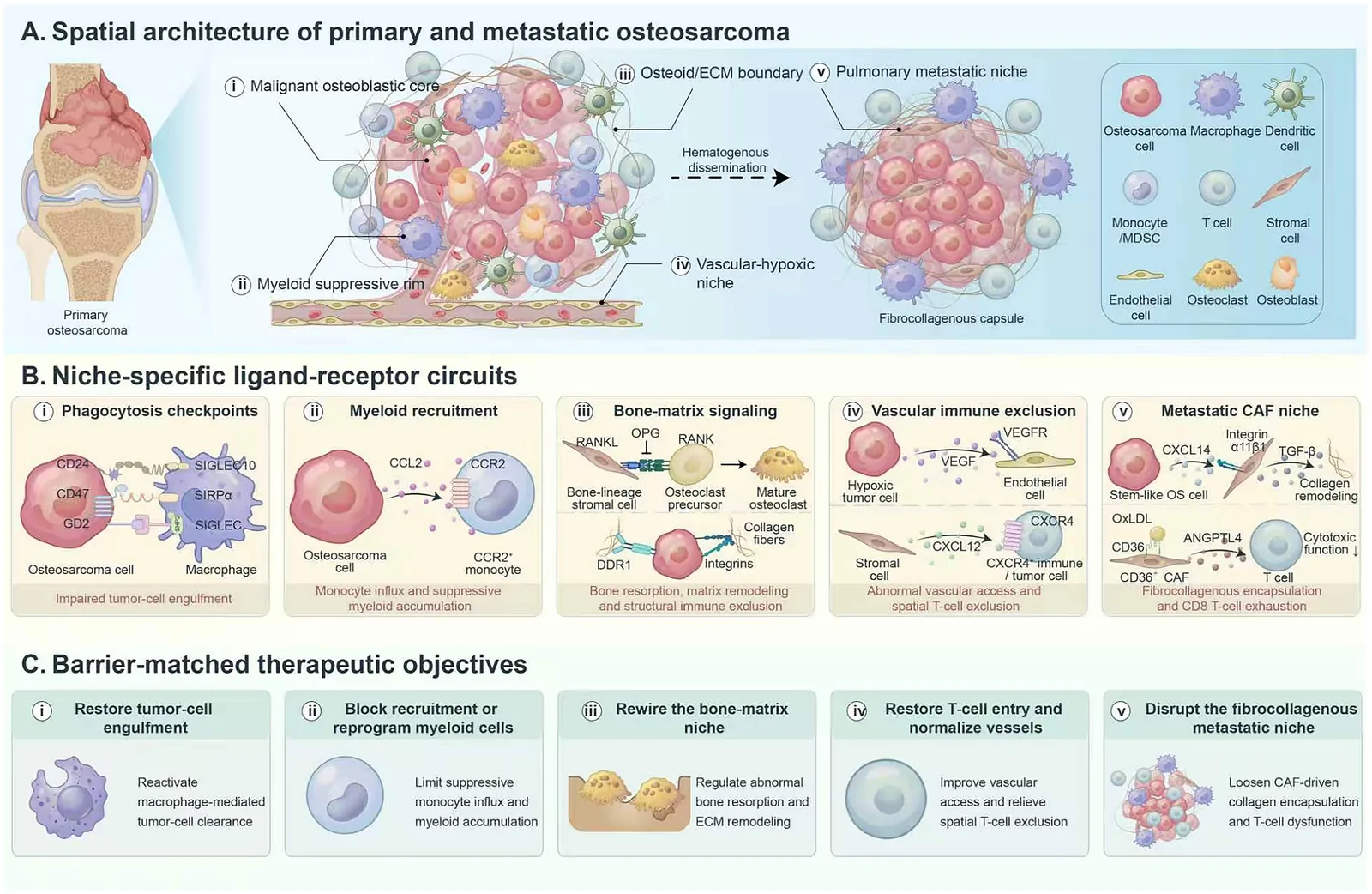
Spatial barriers and candidate communication circuits in osteosarcoma. **(A)** Conceptual organization of primary bone tumors and pulmonary metastases, including tumor–myeloid interfaces, osteoid/extracellular matrix boundaries, vascular regions and fibrocollagenous encapsulation. The illustration synthesizes findings from different cohorts and models; it is not a spatial map measured in one OS dataset. **(B)** Selected communication routes. Lines indicate proposed direction of communication or biological sequence, not a common level of experimental support. In panel B(i), the GD2-associated “SIGLEC” denotes human SIGLEC7 or murine Siglec-E, according to the experimental species; SHP2 is a downstream phosphatase. GD2-receptor perturbation and CD47-associated engulfment have OS experimental support. CD24 perturbation supports an OS anti-phagocytic phenotype, whereas the human SIGLEC10 assignment is computationally inferred in OS. Panel B(ii) combines human/canine CCL2 and migration assays with canine metastatic treatment evidence. In B(iii), RANKL–RANK/OPG represents bone-remodeling biology, and DDR1 has OS tumor-growth evidence; their immune-exclusion consequences remain proposed. In B(iv), vascular immune effects draw on broader cancer biology, while OS CXCR4 findings comprise spatial observations and tumor-cell pharmacology. Panel B(v) brings together two distinct stromal circuits: CXCL14–integrin α11β1 has experimental pulmonary-niche support; OxLDL uptake by CD36-positive CAFs leads to ANGPTL4 signaling through integrin α5β1 on CD8 T cells. The latter circuit was investigated in treated human OS and subcutaneous/paratibial mouse models, not specifically within a demonstrated pulmonary capsule. **Table 1** details the models, assays and endpoints for each route. **(C)** Proposed therapeutic objectives, rather than demonstrated outcomes for every illustrated circuit or established treatment-selection criteria. CAF, cancer-associated fibroblast; ECM, extracellular matrix; MDSC, myeloid-derived suppressor cell; OPG, osteoprotegerin; OS, osteosarcoma; OxLDL, oxidized low-density lipoprotein. **Figure 1** was prepared using SAI and Adobe Illustrator.

**Table 1:** Evidence supporting selected ligand–receptor circuits and microenvironmental barriers in osteosarcoma.

Axis or circuit	Sender → recipient	Model/site	Evidence level	Experimental support	Open biological question	Refs.
CD24–SIGLEC10	OS cell → macrophage	Human OS, mixed lesions; K7M2/BMDM culture and murine periosteal tumor	OS functional: CD24; OS inference: SIGLEC10; other-cancer receptor evidence	CD24 tissue protein; Cd24a knockdown increased engulfment in culture and mice. Human SIGLEC10 dependence not tested in the OS intervention.	Which receptor mediates the OS phenotype?	(13, 21)
CD47–SIRPα	OS cell → macrophage	Human OS tissue/cells; LPS-activated macrophage coculture	OS functional, *in vitro*; broader checkpoint biology	CD47 protein staining and antibody-associated phagocytosis; macrophage activation is part of the experimental condition.	Response in intact bone/lung niches and human efficacy	(22, 23)
GD2–SIGLEC7 (human); GD2–Siglec-E (mouse)	OS cell → macrophage; downstream SHP2	Human specimens/cell assays; murine BMDMs; nude-mouse lung and intratibial models	OS functional + human tissue association	Binding and receptor knockdown; SHP2 phosphorylation; engulfment assays; reduced lung burden with GD2/SHP2 intervention.	Adaptive immune effects; transfer between human and murine receptors	(20)
CSF1–CSF1R; PI3Kγ signaling	Tumor/stroma → macrophage	Other-cancer models; human OS–macrophage–endothelial chip	OS functional, engineered *in vitro*; pan-cancer support	Pexidartinib/tenalisib changed macrophage markers and cytokines on-chip. These are drug effects, not complete ligand–receptor validation.	Selective receptor dependency, T-cell rescue and *in vivo* OS efficacy	(26–28)
CCL2–CCR2	OS cell → monocyte	Human/canine cultures; spontaneous canine pulmonary metastatic OS	OS functional, cross-species; canine treatment study	Secreted CCL2 and migration assays; losartan pharmacodynamics; combination treatment with toceranib in dogs.	Human exposure and efficacy; effects of selective CCR2 blockade	(25)
CXCL12–CXCR4	Stromal compartment → immune/tumor cell	Human pediatric lung metastases; patient-derived OS cultures	OS spatial; tumor-cell pharmacology	Regional expression and protein profiling; CXCR4/MIF inhibition reduced viability, whereas CXCL12 inhibition did not in tested cultures.	Causal T-cell exclusion and its reversal; operative ligand in each region	(11, 29)
CXCL14–integrin α11β1	Stem-like OS cell → fibroblast	Human primary/lung specimens; human fibroblasts and murine lung-niche models	OS functional + human tissue association	Secretion ELISA; recombinant-protein pull-down/co-IP; tissue staining; gel contraction; antibody intervention with immune/metastatic endpoints.	Prospective human response biomarker; generalizability across sites	(30)
OxLDL/CD36 → ANGPTL4–integrin α5β1	Lipid uptake by CAF → secreted ANGPTL4 → CD8 T cell	Treated human OS; human cell assays; K7M2 subcutaneous/paratibial models	OS functional, multistep circuit	CD36 interference; protein secretion; ANGPTL4 pull-down/PLA-flow; α5β1 blockade; T-cell function; mouse combination experiments.	Pretreatment prediction; pulmonary-capsule location; clinical benefit	(31)
MIF–(CD74+CD44) or MIF–(CD74+CXCR4)	OS cell → macrophage	Human OS multiomics/cocultures; subcutaneous K7M2/BALB/c model	OS functional: MIF; OS inference: receptor complexes	MIF protein/secretion, knockout and recombinant rescue; macrophage phenotype/migration; anti-PD-1 combination. Receptor complexes were inferred.	Receptor-specific binding, activation and causal dependency	(32)
RANKL–RANK/OPG	Bone-associated cell → osteoclast lineage	General bone biology; human OS tissue; OS osteolysis models	Bone biology; OS remodeling evidence	OPG is a soluble decoy. OS integrin α2 interventions measured osteoclast-related signaling and bone damage, not immune restoration.	Effect of remodeling on immune access and effector function	(15, 33, 34)
Collagen–DDR1/integrins	ECM → tumor/stromal cell	Human OS tissue; OS cell, clonogenic and 3D cultures	OS functional: DDR1/tumor growth; immune extrapolation	DDR1 IHC, siRNA and inhibitor-associated growth reduction. General ECM biology supports the broader structural hypothesis.	Direct OS evidence for lymphocyte exclusion or rescue	(35, 36)
VEGF–VEGFR; angiopoietin–TIE2	Tumor/stroma → vascular compartment	Pan-cancer vascular studies; human metastatic OS regorafenib trials	Pan-cancer mechanism; OS clinical activity	Vascular biology and randomized progression-free outcomes address different endpoints. Multikinase efficacy does not identify an immune mechanism.	OS vascular normalization linked to immune trafficking and response	(37–40)

Evidence level specifies the claim supported, not a universal ranking of study designs. OS functional evidence can concern tumor growth without establishing immune restoration. Spatial association, protein detection, receptor engagement and perturbation are recorded separately where available. Clinical predictive validation of the candidate spatial biomarkers remains open. BMDM, bone marrow-derived macrophage; CAF, cancer-associated fibroblast; co-IP, co-immunoprecipitation; ECM, extracellular matrix; IHC, immunohistochemistry; LPS, lipopolysaccharide; OS, osteosarcoma; OPG, osteoprotegerin; OxLDL, oxidized low-density lipoprotein; PLA, proximity ligation assay.

## 2 Reading spatial communication across experimental settings

Human primary bone tumors, pulmonary metastases and pleural effusions sample different biological compartments. Primary and mixed-lesion single-cell studies reveal malignant states, myeloid heterogeneity and altered antigen presentation (9, 10, 13). Resected lung metastases provide direct spatial observations of capsule formation, immune-cell segregation and regionally variable chemokine signaling (11, 12). Pleural-fluid cells lack the bone or lung tissue architecture needed to infer a physical exclusion boundary (14). Treatment history also matters: chemotherapy-associated response territories contain different proportions of viable tumor, histiocytes, osteoclasts and extracellular bone matrix (15). Anatomical site and treatment history therefore provide the context for interpreting each communication program.

Spatial localization, protein detection, receptor activation and functional perturbation answer successive questions about an interaction. Tissue maps locate potential partners; binding and activation assays examine receptor engagement; perturbations test the contribution to a phenotype. **Table 1** records these observations alongside the model and anatomical site. CellPhoneDB, NicheNet and CellChat nominate interactions from expression data and prior knowledge, providing candidates for this experimental sequence (16–19).

Interpretation also depends on the endpoint: reduced tumor-cell growth, increased macrophage engulfment and restored CD8 function represent different outcomes. Findings from other cancers supply receptor-level context, whereas OS tissue and intervention studies establish disease-specific localization and function.

### 2.1 Literature selection

This narrative review drew on a PubMed search recorded on 1 July 2026 and targeted updates on 27 September 2026. Searches combined osteosarcoma with single-cell, spatial transcriptomics, ligand-receptor, cell-cell communication, immune microenvironment and named pathway terms, including GD2, SIGLEC7, CD24, CSF1R, CCR2, CXCR4, CXCL14, MIF, ANGPTL4, DDR1 and RANKL. An example query was: osteosarcoma[Title/Abstract] AND (“single-cell” OR “spatial transcriptomics” OR “ligand-receptor” OR “cell-cell communication”). Selection favored original OS studies reporting tissue localization, protein assays or perturbation with immune endpoints. Studies of other cancers were used for defined mechanistic context; general reviews supported background and methodological interpretation. Selection was purposive, without a systematic-review protocol or quantitative pooling.

## 3 Tumor–macrophage contact and myeloid recruitment

### 3.1 Phagocytosis and receptor dependence

The GD2 study connects a tumor-surface glycolipid to macrophage behavior through several experimental steps. Binding assays and receptor knockdown implicated SIGLEC7 in human macrophages and Siglec-E in mouse macrophages; downstream experiments linked murine Siglec-E to SHP2 phosphorylation and reduced engulfment (20). GD2 depletion and combined anti-GD2/SHP2 inhibition reduced pulmonary metastatic burden in nude-mouse models. The intratibial model also showed reduced lung dissemination without a comparable effect on primary tumor growth (20). The effect on lung dissemination, together with the engulfment assays, supports a role for macrophage-mediated control of metastasis; adaptive immune effects remain untested in these T-cell-deficient models.

CD24 illustrates why ligand-level and receptor-level evidence must be distinguished. Liu and colleagues combined human single-cell and tissue analyses with Cd24a knockdown in K7M2 cells, macrophage phagocytosis assays and a murine periosteal tumor model (13). These experiments support an anti-phagocytic role for CD24 in OS. The CD24–SIGLEC10 pairing, however, was nominated computationally; the same study did not establish human SIGLEC10 dependence through receptor-specific perturbation. Direct evidence for that receptor mechanism comes from other cancer models, including breast and ovarian cancer (21).

CD47–SIRPα has a broader mechanistic foundation (22). An OS study demonstrated increased engulfment when CD47 antibody treatment was combined with lipopolysaccharide-activated macrophages, linking the response to the macrophage activation state (23). Anti-GD2/CD47 combinations have also shown activity in preclinical models (24). Their relative efficacy and tolerability in patients with OS remain to be determined.

### 3.2 Recruitment and macrophage state

CCL2-dependent recruitment is supported by human and canine OS cell assays, including secreted-protein measurements and monocyte migration experiments (25). The translational treatment study involved dogs with spontaneous pulmonary metastatic OS receiving losartan and toceranib. Pharmacodynamic inhibition of monocyte migration required exposures above conventional antihypertensive dosing (25). These findings provide canine treatment and cross-species migration evidence, with human dose requirements and the contribution of each drug still unresolved.

CSF1R-directed macrophage reprogramming was established initially in other cancer settings, including pancreatic models (26, 27). More recent OS-specific evidence comes from a perfused tri-culture chip containing human OS cells, blood-derived macrophages and endothelial cells. Pexidartinib and tenalisib, studied as interventions in CSF1R and PI3Kγ signaling, altered macrophage-associated gene expression and cytokine profiles in this system (28). This engineered system supports pharmacological modulation of macrophage state. Receptor-selective effects and downstream T-cell responses are the next mechanistic questions.

## 4 Chemokine patterns at the pulmonary interface

OS immune profiling identifies heterogeneous chemokine communication programs (29). In pediatric pulmonary metastases, spatial analysis found different CXCR4-associated ligand patterns inside and outside tumor regions (11). The accompanying cell-line experiments are especially informative: CXCR4 and MIF inhibition reduced tumor-cell viability, whereas CXCL12 inhibition did not produce a comparable effect in the tested cultures. The viability results point to ligand-dependent differences in tumor-cell responses, while the spatial data locate candidate communication zones. A role in T-cell exclusion remains a separate hypothesis (11).

CXCL14 provides a more direct link between tumor-derived signaling and fibroblast-mediated niche formation. Xu and colleagues identified CXCL14 enrichment in a stem-like malignant population and higher expression in lung metastases than in primary tumors (30). Their experimental approach separated ligand secretion, measured in culture supernatants by ELISA, from receptor binding, tested by recombinant-protein pull-down and co-immunoprecipitation. Multiplex staining localized integrin α11 and β1 to α-SMA-positive fibroblasts; collagen-gel contraction assays assessed the resulting stromal response. The study reported increased actomyosin contractility, matrix remodeling and fibroblast TGF-β production. Antibody interference reduced TGF-β production, enhanced CD8-mediated antitumor activity and suppressed experimental lung metastasis (30). Together, the human tissue comparisons, binding assays and intervention models connect ligand production to stromal activation and experimental pulmonary immune suppression.

## 5 Fibroblast metabolism and bone-associated barriers

### 5.1 Metabolic signals that impair immune function

A study of chemotherapy/anti-PD-1-treated OS linked CD36-positive cancer-associated fibroblasts (CAFs) to oxidized low-density lipoprotein (OxLDL) uptake and ANGPTL4 secretion (31). CD36 interference reduced lipid uptake, while downstream analyses implicated a PPARG–FABP4 program. In human CD8 T-cell experiments, ANGPTL4 pull-down and proximity ligation supported interaction with integrin α5β1; receptor blockade alleviated cytokine suppression and apoptosis. Vitamin E enhanced anti-PD-1 activity in subcutaneous and paratibial K7M2 mouse models (31). Lipid uptake by CAFs is thus linked to receptor-mediated suppression of neighboring T cells in a circuit supported across treated human tumors and experimental local-tumor settings.

Tumor-derived macrophage migration inhibitory factor (MIF) represents a separate route to myeloid dysfunction. OS-cell MIF knockout and recombinant-MIF rescue changed macrophage polarization and migration in coculture; combined MIF inhibition and PD-1 blockade improved tumor control in a subcutaneous K7M2 model (32). By contrast, MIF–(CD74+CD44) and MIF–(CD74+CXCR4) were identified through LR inference. MIF-dependent macrophage effects are therefore experimentally supported, while the relative contribution of the two receptor complexes remains unresolved.

The two metabolic studies also differ in the information supplied by human tissue. The CAF study sampled tumors after chemotherapy and anti-PD-1 treatment, whereas the MIF work integrated metabolic and transcriptomic risk groups with experimental cocultures (31, 32). Post-treatment stromal states can reflect selection or adaptation. Their value for patient selection depends on whether the same features are present before treatment and predict differential benefit from an intervention.

### 5.2 Matrix remodeling and osteoclast activity

Bone remodeling creates a different setting from fibrocollagenous encapsulation in the lung. RANKL–RANK signaling governs osteoclast differentiation, while osteoprotegerin (OPG) acts as a soluble decoy receptor (33). OS imaging studies show that osteoclasts, histiocytes and matrix occupy heterogeneous response territories (15). An integrin α2 study linked OS-cell perturbation to osteoclast-related signaling and osteolytic damage in mouse models (34). The demonstrated outcome is bone destruction; its consequences for immune-cell entry and antigen handling remain to be established.

Collagen can provide both a physical matrix and a receptor stimulus (35). OS-specific DDR1 studies include protein expression in tumor specimens and reduced tumor-cell growth after siRNA or pharmacological inhibition (36). The OS evidence concerns tumor-cell growth. Collagen-dependent lymphocyte exclusion remains a proposed immune consequence, informed by broader matrix biology. In this setting, bone remodeling and collagen sensing describe microenvironmental processes rather than receptor-mediated immune checkpoints.

The connection to immunity lies in the changing tissue interface. Digital pathology can relate matrix-rich and osteolytic territories to immune-cell distribution (15). Combining these measurements with tumor–immune contact and effector function would clarify whether remodeling alters access to malignant cells independently of changes in tumor burden.

## 6 Vascular signaling and the meaning of clinical activity

VEGF–VEGFR and angiopoietin–TIE2 signaling regulate vascular organization and leukocyte access across cancer models (37, 38). In OS, the vascular hypothesis links regional hypoxia and abnormal vessels to impaired lymphocyte access. Measurements of endothelial pathway activity and transendothelial trafficking would distinguish this mechanism from poor recognition or suppression after tissue entry.

Regorafenib improved progression-free outcomes in randomized phase 2 studies of metastatic OS (39, 40). The trials establish clinical antitumor activity, with the contribution of vascular normalization or immune restoration undetermined. That distinction is central to interpreting investigational combinations with checkpoint blockade. Paired measurements of vessel perfusion, hypoxia, endothelial activation and extravascular CD8 localization could help test the proposed mechanism, particularly if obtained before substantial tumor shrinkage changes tissue architecture.

## 7 From spatial measurements to treatment hypotheses

Spatial biomarkers could link a cellular dependency to a measurable tissue feature. Candidate measurements include the fraction of receptor-positive macrophages contacting GD2-positive tumor cells, the proportion of CD8 cells inside versus outside viable tumor nests, and the continuity or thickness of a fibrocollagenous boundary. For the CAF metabolic circuit, a composite measurement could combine CD36/ANGPTL4-positive fibroblasts with neighboring integrin α5β1-positive CD8 cells and a functional T-cell readout (20, 31). Prospective validation would determine whether these candidate features predict treatment benefit.

Validation requires lesion-level sampling with patient-level analysis. Pretreatment and on-treatment tissue should be compared within the same anatomical setting, with necrosis, prior chemotherapy and metastatic status recorded. A marker associated with survival is not necessarily predictive of benefit from its matched intervention (12). Predictive use requires testing whether treatment effects differ by marker status and then confirming that relationship in an independent population. Where repeated biopsies are impractical, an early pharmacodynamic study may establish target engagement without yet supporting patient selection.

The measured feature should also match the proposed intervention. Recruitment can be examined through ligand-producing cells and incoming receptor-positive monocytes; total macrophage density loses that distinction. Phagocytosis requires tumor–macrophage contact and an engulfment readout. For vascular mechanisms, separating lymphocytes retained within vessels from those that have crossed the endothelium would make the spatial analysis more informative than a pooled CD8 count.

Feasibility also constrains combinations. CD47-directed therapy can affect normal blood cells, and macrophage interventions may disturb clearance or inflammatory homeostasis (22). Chemokine blockade alters trafficking beyond the tumor (25). Bone-remodeling interventions intersect with skeletal maintenance, while antiangiogenic treatment can complicate vascular function and wound repair (33, 37). These concerns favor measuring both the intended immune effect and normal-tissue consequences, especially around orthopedic surgery or pulmonary metastasectomy.

## 8 Discussion

The strongest mechanistic findings connect a defined cellular interaction to a change in immune function. In OS, tumor-surface control of phagocytosis and fibroblast-mediated T-cell dysfunction provide two such examples (13, 20, 31). Their anatomical coverage remains uneven, however. Comparing these dependencies in bone and lung could reveal whether dissemination preserves an immune-evasion program or exposes a new vulnerability in the metastatic niche.

Spatial LR inference has identifiable technical limits. Transcript detection does not measure secreted ligand availability, cell-surface protein abundance, receptor occupancy or pathway activation (19). Dissociation removes location, while multicellular spatial spots can merge sender and receiver identities. Predicted direction follows the interaction database and cell annotations; it is not a direct observation of signal flow. Diffusible ligands also make a single distance cutoff inappropriate for every interaction. Protein imaging and downstream phosphoprotein assays can narrow these uncertainties, but causal assignment still requires perturbation. In bone, decalcification, tissue preservation and chemotherapy-related necrosis additionally affect which compartments and markers can be assessed (9, 15).

Model choice can help resolve these gaps. A macrophage coculture is useful for testing receptor-dependent engulfment, whereas evaluation of T-cell recruitment requires a system with functional lymphocytes and an accessible tissue compartment. Orthotopic and lung-colonization models answer different questions about establishment and maintenance of a niche. Within each system, immune depletion or cell-specific perturbation can test whether tumor control depends on the proposed recipient cell. This would distinguish a genuine immune contribution from a drug effect on malignant cells, a distinction left unresolved by several of the current studies.

Three questions are experimentally tractable. First, does receptor-specific loss and rescue reproduce the immune effects attributed to CD24 or MIF? Second, do matched bone and lung models preserve the same cellular dependency after treatment? Third, does disrupting a candidate circuit improve immune function and tumor control together, rather than only reducing tumor-cell viability? Answering these questions would connect the existing spatial observations to mechanisms that can be tested in patients.
